# Herbicolin A production and its modulation by quorum sensing in a *Pantoea agglomerans* rhizobacterium bioactive against a broad spectrum of plant‐pathogenic fungi

**DOI:** 10.1111/1751-7915.14193

**Published:** 2022-12-18

**Authors:** Miguel A. Matilla, Terry J. Evans, Jesús Martín, Zulema Udaondo, Cristina Lomas‐Martínez, Míriam Rico‐Jiménez, Fernando Reyes, George P. C. Salmond

**Affiliations:** ^1^ Department of Biotechnology and Environmental Protection, Estación Experimental del Zaidín Consejo Superior de Investigaciones Científicas Granada Spain; ^2^ Department of Biochemistry University of Cambridge Cambridge UK; ^3^ Fundación MEDINA Centro de Excelencia en Investigación de Medicamentos Innovadores en Andalucía Granada Spain; ^4^ Department of Biomedical Informatics University of Arkansas for Medical Sciences Little Rock Arkansas USA

## Abstract

Global population growth makes it necessary to increase agricultural production yields. However, climate change impacts and diseases caused by plant pathogens are challenging modern agriculture. Therefore, it is necessary to look for alternatives to the excessive use of chemical fertilizers and pesticides. The plant microbiota plays an essential role in plant nutrition and health, and offers enormous potential to meet future challenges of agriculture. In this context, here we characterized the antifungal properties of the rhizosphere bacterium *Pantoea agglomerans* 9Rz4, which is active against a broad spectrum of plant pathogenic fungi. Chemical analyses revealed that strain 9Rz4 produces the antifungal herbicolin A and its biosynthetic gene cluster was identified and characterized. We found that the only acyl‐homoserine lactone‐based quorum sensing system of 9Rz4 modulates herbicolin A gene cluster expression. No role of plasmid carriage in the production of herbicolin A was observed. Plant assays revealed that herbicolin A biosynthesis does not affect the root colonization ability of *P. agglomerans* 9Rz4. Current legislative restrictions are aimed at reducing the use of chemical pesticides in agriculture, and the results derived from this study may lay the foundations for the development of novel biopesticides from rhizosphere microorganisms.

## Graphical abstract

Herbicolin A biosynthesis is responsible for the strong antagonistic activities of the rhizosphere bacterium *Pantoea agglomerans* 9Rz4 against a broad spectrum of phytopathogenic fungi.

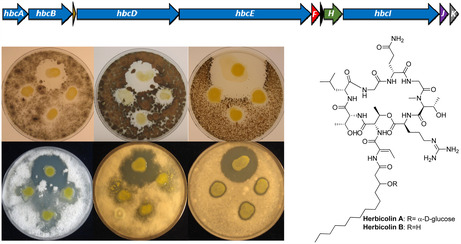

## INTRODUCTION

The plant microbiome, defined as the community of microorganisms associated with their plant hosts (Cordovez et al., 2019), plays an essential role in plant growth and protection against plant pathogens (Trivedi et al., 2020). Thus, plants are considered as holobionts consisting of the plant host and its associated microbiota (Bakker et al., 2020; Trivedi et al., 2020). This interaction is highly dynamic and involves a complex network of signalling molecules (Berlanga‐Clavero et al., 2020; Matilla & Krell, 2018; Trivedi et al., 2020). In fact, plants have been shown to select specific microorganisms and microbial functions to counteract different biotic and abiotic stresses, for example, improving nutrient acquisition and disease resistance (Bakker et al., 2020; Trivedi et al., 2020). The ‘cry for help’ hypothesis supports the notion that plants can actively recruit beneficial microorganisms to help them to cope against stresses, including protection against plant pathogens (Rizaludin et al., 2021). For example, it was shown that sugar beet plants recruit specific endophytic microorganisms upon infection with the fungal phytopathogen *Rhizoctonia solani* (Carrión et al., 2019).

Protective plant microbiomes have been associated with the presence of gene clusters for the biosynthesis of secondary metabolites. Comparative metagenomic analyses of the endosphere of plants have revealed an enrichment in secondary metabolite biosynthetic clusters in endophytes from plants grown in disease suppressive soils compared with plants growing in disease conductive soils (Carrión et al., 2019). In addition, the genetic potential for the production of secondary metabolites has been analysed in the *Populus* root microbiome, leading to the identification of a diversity of natural product gene clusters (Blair et al., 2018). Furthermore, genome mining approaches also highlighted a strong secondary biosynthetic potential in bacterial phyllosphere isolates (Helfrich et al., 2018). Such studies reveal the enormous potential of plant microbiomes for the identification and isolation of new bioactive compounds.

Polyketides (PKs) and non‐ribosomal peptides (NRPs) are two of the largest and most diverse families of secondary metabolites. These metabolites are synthesized by polyketide synthases (PKSs) and nonribosomal peptide synthetases (NRPSs), respectively, through sequential rounds of condensation of malonyl‐coA and amino acid extension units (Little & Hertweck, 2022). PKs and NRPs have a broad range of biological activities, including antibacterial, antifungal, antiviral, herbicidal, immunosuppressive, anticancer and other functions (Little & Hertweck, 2022; Sussmuth & Mainz, 2017). This diversity of bioactivities resides frequently in the chemical modifications carried out on PKs and NRPs backbones by various tailoring enzymes (e.g. halogenases, hydroxylases, oxidases, glycosylases, etc.) (Little & Hertweck, 2022; Sundaram & Hertweck, 2016; Sussmuth & Mainz, 2017).

Genome mining approaches have estimated that only approximately 3% of the bacterial secondary biosynthetic potential has been assessed experimentally (Gavriilidou et al., 2022) and Enterobacteria, including those that live in association with plants, are among the taxonomic groups with the highest biosynthetic potential (Gavriilidou et al., 2022; Mohite et al., 2022). PKS and NRPS gene clusters were found among the most abundant secondary metabolite gene clusters in plant‐associated bacteria (Blair et al., 2018; Carrión et al., 2019; Helfrich et al., 2018). Some examples of bioactive PKs and NRPs produced by plant‐associated Enterobacteria include the antifungal and anti‐oomycete polyketide, oocydin A (Matilla et al., 2012), the hybrid non‐ribosomal peptide‐polyketide antimicrobials, andrimid (Matilla et al., 2016), serratamid (Nguyen et al., 2021) and solanimycin (Matilla et al., 2022), and the hybrid non‐ribosomal peptide‐polyketide toxin, zeamine (Hellberg et al., 2015).

Previous work on rhizosphere organisms from different plants of agricultural relevance led to identification of diverse enterobacterial species with the capacity to inhibit the growth of the plant pathogenic fungus *Verticillium dahliae* (Berg et al., 2002). Among more than 5800 rhizosphere isolates initially characterized, the oilseed rape rhizosphere bacterium *Pantoea agglomerans* 9Rz4 was one of the most efficient bacterial isolates antagonizing fungal growth—being also highly bioactive against *Rhizoctonia solani* and *Sclerotinia sclerotiorum* (Berg et al., 2002). Here, a combination of genetic, genomic and analytical chemistry approaches enabled the characterization of fungal antagonism mechanisms in the strain 9Rz4. The antifungal biosynthetic gene cluster was identified and the role of quorum sensing and plasmid carriage in the regulation of the production of this antifungal antibiotic was also interrogated.

## RESULTS AND DISCUSSION

### Isolation of non‐bioactive mutants of *Pantoea agglomerans* 9Rz4

**FIGURE 1:**
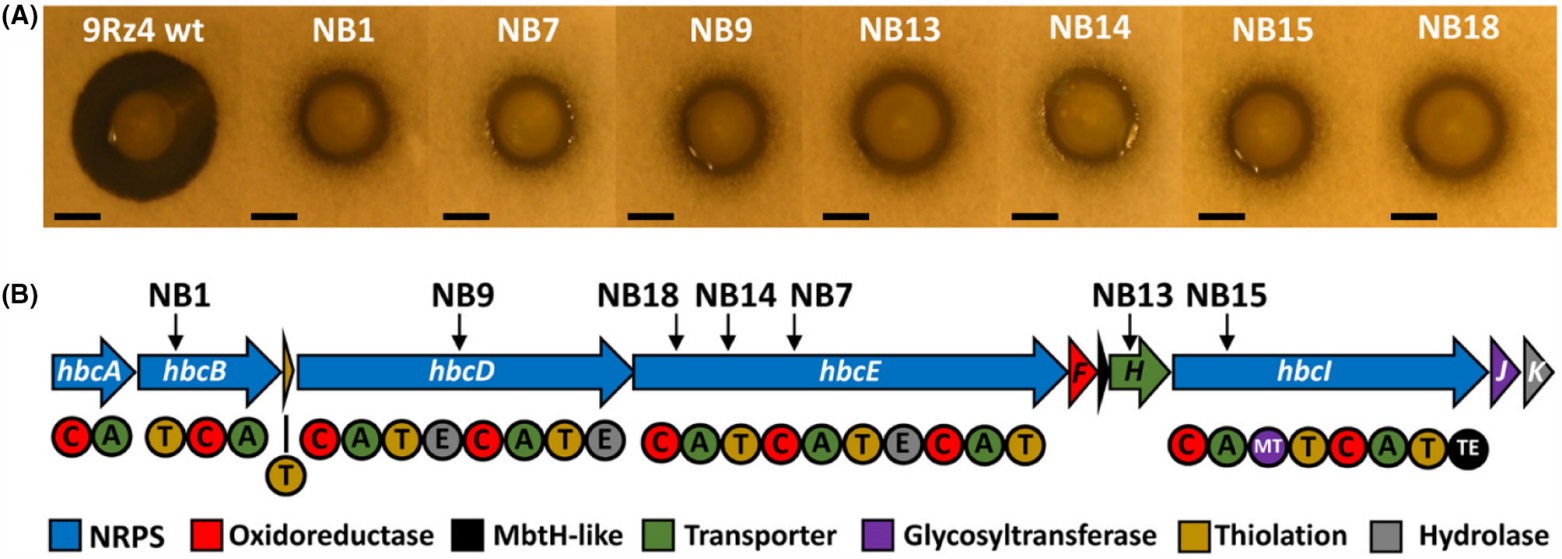
Identification and characterization of a novel NRPS antifungal gene cluster in *Pantoea agglomerans* 9Rz4. (A) Bioactivities against *V. dahliae* of 9Rz4 and derivative mutant strains. The antagonistic bioassays were conducted as described in Matilla et al. (2012). *V. dahliae* is present in the top agar lawns and the size of the inhibition halo determines its susceptibility to the antifungal antibiotic herbicolin A. These assays were repeated at least three times and representative results are shown. The inhibition halo radius measured for the 9Rz4 wild‐type strain was 4.4 ± 0.2 mm (data from three biological replicates). Pictures were taken after 96 h of incubation at 25°C. The generalized transducing phage ϕOT8 was used for transduction of chromosomal mutations, as previously described (Evans et al., 2010). Transductants were selected on plates containing kanamycin, and retention of phage sensitivity was confirmed in the transductants. Bars, 5 mm. (B) Genetic organization of the herbicolin A gene cluster in 9Rz4, which spans from *NO300_21850* (*hbcA*) to *NO300_21800* (*hbcK*). Location of the transposon insertions are indicated by black arrows with the indicated strain name above. Colour code representing the functional category of each gene of the biosynthetic cluster. A, AMP‐binding; C, condensation; E, epimerization; MT, methyltransferase; T, thiolation; TE, thioesterase.

To identify the genes involved in the biosynthesis of the unknown antifungal antibiotic, a random mutagenesis approach using the transposon Tn‐KRCPN1 (Monson et al., 2015) was employed as a strategy to generate a *P. agglomerans* 9Rz4 mutant library. The strain 9Rz4 proved to be genetically tractable and the phenotypic characterization of ~2500 random transposon mutants in dual plate bioassays enabled the isolation of seven independent transposon mutants (non‐bioactive (NB) strains NB1, NB7, NB9, NB13, NB14, NB15 and NB18) which were defective in their antagonistic activities against *V. dahliae* (Figure 1A). To confirm that the loss of antifungal activity was caused by single insertions, the flagellum‐dependent generalized transducing phage ϕOT8 (Evans et al., 2010) was used to transduce the transposon insertions back into the wild‐type strain 9Rz4. Bacteria, oomycete, fungi, phages and plasmids used in this study are listed in Table S1. All the resulting transductants had the same phenotypes as the parental mutants, thereby confirming that each individual insertion caused loss of the bioactivity against *V. dahliae*. Random primed PCR was conducted to identify the location of the transposon insertions, using methods previously described (Monson et al., 2015) and using oligonucleotides detailed in Table S1. The insertions mapped to several genes encoding NRPSs and BLAST analyses revealed low homology to sequences available in the NCBI database—suggesting an uncharacterized antifungal biosynthetic gene cluster.

### Herbicolin A is the antifungal antibiotic produced by 9Rz4

**FIGURE 2:**
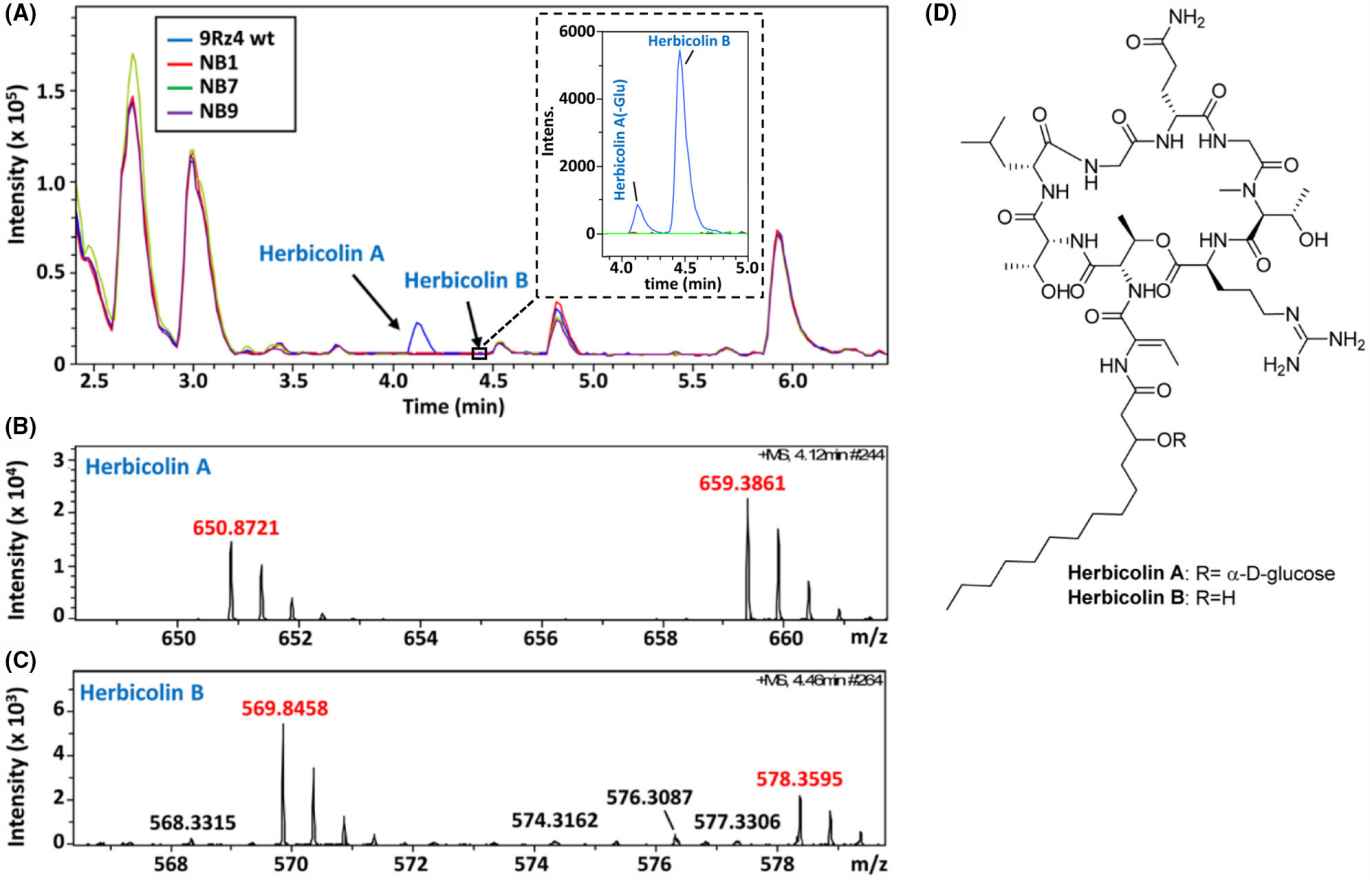
Identification of herbicolins A and B in *Pantoea agglomerans* 9Rz4 supernatants by liquid chromatography–high‐resolution mass spectrometry (LC‐HRMS). (A) Base peak chromatogram of the LC‐HRMS analysis of the supernatants of wild‐type 9Rz4 and mutants in the herbicolin A biosynthetic cluster (e.g. NB1, NB7 and NB9). The insert shows a zoomed region of the extracted ion chromatogram corresponding to an *m*/*z* of 569.8452 ± 0.0050 (theoretical value for [M + 2H]^2+^ in herbicolin B), confirming the presence of herbicolin B at 4.46 min in the supernatant of wild‐type 9Rz4 and its absence in those supernatants of mutants in the herbicolin A biosynthetic cluster. The peak at 4.12 min is originated by fragmentation of herbicolin A, with loss of the glucose unit. (B) Expansion of the HRMS spectrum of herbicolin A. (C) Expansion of the HRMS spectrum of herbicolin B. In (A–C), 5 μl supernatants of bacteria grown in minimal medium with glucose as sole carbon source was used for the LC‐HRMS analyses. (D) Chemical structures of herbicolins A and B.

To identify the antifungal compound synthesized by 9Rz4, we cultured the wild‐type bacteria and several mutants deficient in their antifungal activities in different media, including minimal medium (Matilla et al., 2012), lysogeny broth (LB), potato dextrose broth (e.g. Strobel medium [Strobel et al., 1999]) and YE broth. Subsequently, dual culture bioassays were conducted to evaluate the in vitro antifungal activities of cell‐free supernatants. The highest antifungal levels in the wild‐type strain were seen when grown in LB, YE and in minimal medium with glucose as sole carbon source (Figure S1). No antifungal activity was detected in the supernatants of any of the NB mutants tested (Figure S1). We subsequently analysed the culture supernatants of *P. agglomerans* 9Rz4 wild‐type strain as well as those of the non‐bioactive mutants NB1, NB7 and NB9 by liquid chromatography–high resolution mass spectrometry (LC‐HRMS) using an Agilent 1200 Rapid Resolution HPLC interfaced to a Bruker maXis mass spectrometer, using the conditions described previously (Martín et al., 2014). One differential metabolite, herbicolin A, was identified in the wild type but not in the NB1, NB7 and NB9 supernatants based on the presence of a peak with ions in its HRMS at *m*/*z* 650.8721 ([M + 2H]^2+^, Calcd 650.8716) and 659.3861 ([M + H + NH_4_]^2+^, Calcd. 659.3848) (Figure 2A,B), consistent with a molecular formula of C_58_H_101_N_13_O_20_. The structurally related herbicolin B, identified as a deglycosylated intermediate during the biosynthesis of herbocilin A (Xu et al., 2022), was also detected at trace levels in the wild type but not in the NB mutant strains, based on the presence of HRMS peaks at *m*/*z* 569.8458 ([M + 2H]^2+^, Calcd. 569.8452) and 578.3595 ([M + H + NH_4_]^2+^, Calcd 659.578.3584) (Figure 2A,C), corresponding to a molecular formula of C_52_H_91_N_13_O_15_. Herbicolins A and B (Figure 2D) are different from another antibiotic known as herbicolin I (also named dapdiamide) that is produced by the biocontrol bacterium *P. vagans* C9‐1. Herbicolin I has antibacterial activities against the plant pathogen *Erwinia amylovora* (Anderson et al., 2004) and has a chemical formula of C_12_H_21_N_4_O_6_ (Kamber et al., 2012).

Herbicolins A and B were originally isolated from *Erwinia herbicola*, now reclassified as *Pantoea agglomerans*, in the 1980s due to their antifungal activities against sterol‐containing fungi (Winkelmann et al., 1980). In contrast to herbicolin I (Anderson et al., 2004), no antibacterial activity was detected for herbicolins A and B (Winkelmann et al., 1980). Subsequent studies revealed that herbicolin A also exhibits activity against *Mycoplasma*, *Ureaplasma* and *Acholeplasma* cells (Birkelund et al., 1986).

### Identification of the herbicolin A biosynthetic gene cluster

Although herbicolins A and B were identified over 40 years ago (Winkelmann et al., 1980), at the start of these investigations there were no reports on the corresponding gene cluster and their biosynthetic route. However, their structures, like those of other bacterial lipopeptides (Baltz, 2014; Girard et al., 2020), suggest biogenesis from a nonribosomal peptide synthase‐type origin. Previous work on the bacterium *Erwinia herbicola* CHS1065 correlated genetic potential for herbicolin A synthesis with possession of the 170 kb plasmid pHER1065 (Tenning et al., 1993), of unknown sequence.

To identify and characterize the biosynthetic gene cluster responsible for the production of herbicolin A in *P. agglomerans* 9Rz4, its genome was sequenced by MicrobesNG® using their enhanced approach, which combines Illumina short reads and Oxford Nanopore technologies sequencing (long reads) in order to obtain high‐quality full genomes. Illumina sequencing generated 1,088,798 paired‐end shorts reads with a sequencing depth of 106×, whereas Oxford Nanopore sequencing libraries were prepared using the SQK‐RBK004 kit and SQK‐LSK109 kit with Native Barcoding EXP‐NBD104/114 (ONT, United Kingdom) using 400–500 ng of high molecular weight DNA. Barcoded Oxford Nanopore samples were pooled into a single sequencing library and loaded in a FLO‐MIN106 (R.9.4.1) flow cell in a GridION (ONT, United Kingdom) generating a total of 6875 reads with a mean length of 10,526 kb and a mean per‐sequence quality score of Q19. Short reads were adapter‐trimmed using Trimmomatic 0.30 with a sliding window quality cutoff of Q15 (Bolger et al., 2014) and a hybrid genome assembly was performed using Unicycler v0.4.0 (Wick et al., 2017). The enhanced assembly resulted in 11 contigs larger than 1000 bp. The largest contig was 3,899,485 bp. The genome of 9Rz4 contains one chromosome and four plasmids of 536,203 kb (p9Rz4_1), 177,238 kb (p9Rz4_2), 142,672 kb (p9Rz4_3) and 59,924 kb (p9Rz4_4) in size (GenBank accession number of the genome of *P. agglomerans* 9Rz4: JANHBZ000000000). Plasmids p9Rz4_1 and p9Rz4_2 are highly homologous to plasmids pPagL15_1 (GenBank: NZ_CP034149.1) and pPagL15_2 (GenBank: NZ_CP034150.1) from *Pantoea agglomerans* L15 respectively. However, p9Rz4_3 and p9Rz4_4 showed low sequence homology with other plasmids deposited in the NCBI database (NCBI Resource Coordinators, 2016).

The availability of the genome of 9Rz4 enabled facile mapping of all the Tn‐KRCPN1 transposon insertions to a 38.6 kb uncharacterized NRPS gene cluster located in the p9Rz4_3 plasmid (Figure 1B). A very similar ~38 kb biosynthetic gene cluster (63.5% identical) was identified in the plasmid p5D of *Candidatus* Fukatsuia symbiotica 5D (Patel et al., 2019), a bacterium isolated from the pea aphid *Acyrthosiphon pisum*. Consequently, the upstream and downstream ends of the herbicolin A gene cluster of *P. agglomerans* 9Rz4 were assigned based on this homology.

The herbicolin A biosynthetic cluster of 9Rz4 is composed of 11 genes, named *hbcA* to *hbcK*, encoding five multifunctional NRPSs (HbcA, HbcB, HbcD, HbcE and HbcI) which include a total de 9 condensation (C), 9 AMP‐binding (A), 9 thiolation (T), 3 epimerization (E), 1 methyltransferase (MT) and 1 thioesterase (TE) domains (Figure 1B). A free‐standing thiolation protein (HbcC) was identified. The herbicolin A cluster also encodes an oxidoreductase (HbcF), a glycosyltransferase (HbcJ) and a hydrolase (HbcK) (Figure 1B). A putative transporter (HbcH), possibly involved in the extrusion of herbicolins A and B to the extracellular environment, was encoded in the biosynthetic gene cluster (Figure 1B). A MbtH‐like protein (HbcG) was also identified. MbtH proteins are small and their function remains unknown, but it has been shown that they interact with adenylation domains of NRPSs to modulate amino acid activation (Bernhardt et al., 2020; Felnagle et al., 2010).

During the drafting of this article, the sequence of a gene cluster responsible for the production of herbicolin A in *Pantoea agglomerans* ZJU23, a bacterium isolated from perithecia formed by *Fusarium graminearum*, was identified (Xu et al., 2022). This gene cluster is highly homologous (91.5% identical) to the herbicolin A cluster of 9Rz4 and is located on a 171 kb plasmid. However, some differences were found between both clusters: (i) the biosynthetic cluster ZJU13 consist of 10 open reading frames (ORFs; named *acbA* to *acbJ*), whereas the homologous cluster of 9Rz4 has 11 ORFs, as described above; and (ii) the NRPS‐encoding genes *acbA* and *acbB* in the ZJU13 herbicolin A cluster are divided in three genes in 9Rz4, namely *hbcA*, *hbcB* and *hbcC*; although the same domain arrangement was observed between the contiguous biosynthetic proteins of the herbicolin A gene clusters of 9Rz4 and ZJU23 (Figure 1B) (Xu et al., 2022).

Interestingly, remnants of transposases were identified bordering the herbicolin A gene cluster of 9Rz4, suggesting that this biosynthetic cluster was acquired by horizontal gene transfer. Several additional observations support this hypothesis: (i) the G + C content of the herbicolin A cluster of 9Rz4 (59.5%) is considerably higher than the G + C content of the plasmid in which it is localized, p9Rz4_3 (48.9%) and (ii) the herbicolin A biosynthetic cluster is present in phylogenetically distant bacteria, namely strains belonging to the *Pantoea* genus and the candidatus genus *Fukatsuia* (Patel et al., 2019).

In a previous study, we were able to mobilize by transduction the complete 77 kb oocydin A gene cluster at high frequencies (Matilla & Salmond, 2014). The highly efficient generalized transducting phage ϕOT8 infects *Serratia* and *Pantoea* species, including 9Rz4, as described above (Evans et al., 2010), consistent with the view that this and related phages may be a route for the intra‐ and intergeneric dissemination of the herbicolin A gene cluster.

### Herbicolin A is active against a broad range of fungal phytopathogens and its production does not affect root colonization by *P. agglomerans* 9Rz4

**FIGURE 3:**
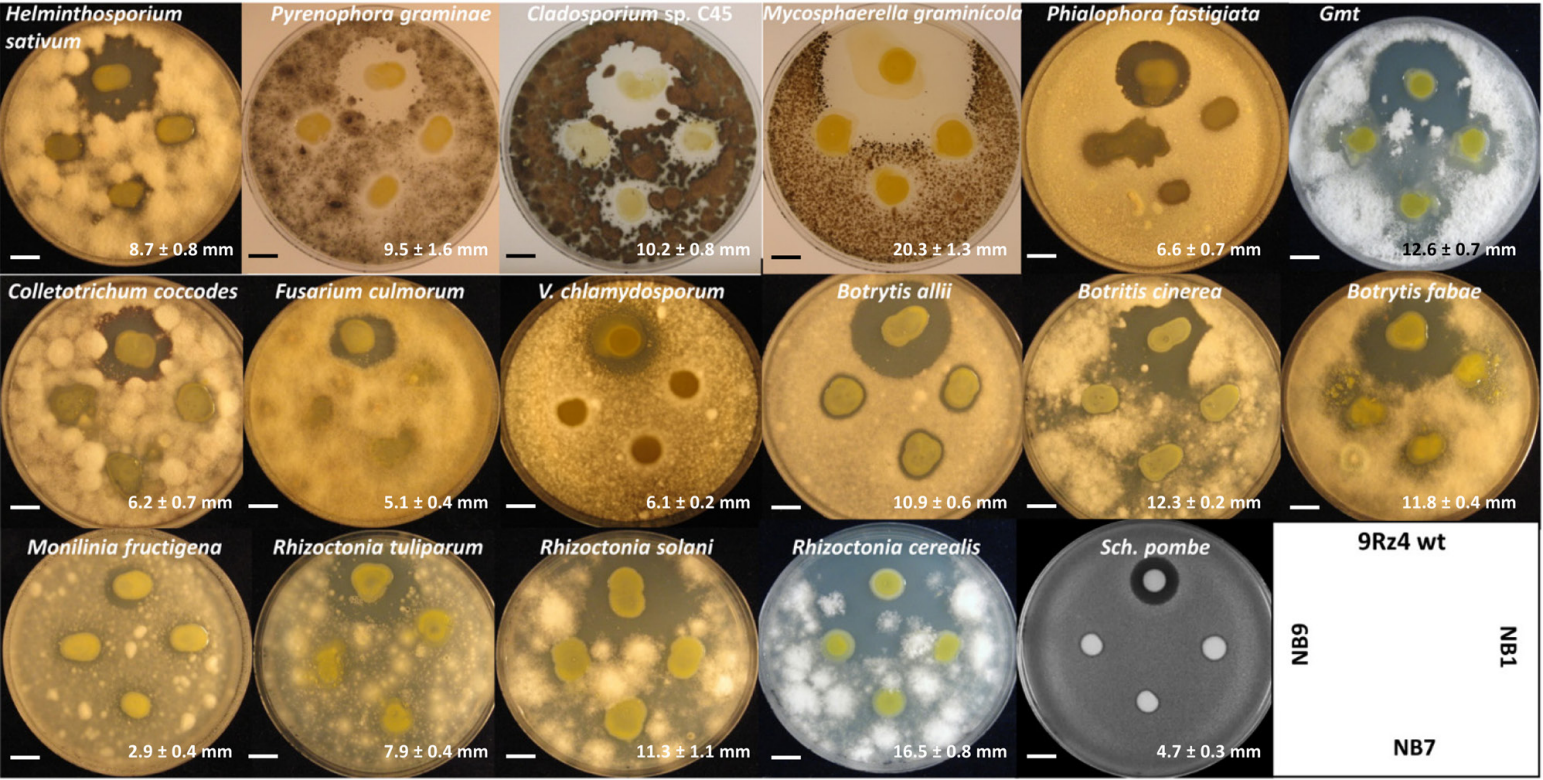
Antifungal activities of herbicolins A and B. Bioactivities of *Pantoea agglomerans* 9Rz4 and derivative mutant strains defective in the herbicolin A gene cluster (e.g. strains NB1, NB7 and NB9) are shown. The bioassays were conducted as previously described (Matilla et al., 2012). Fungi are present in the top agar lawns and the size of the inhibition halo determines their susceptibility to herbicolin A produced by 9Rz4. All the fungi strains tested in this study are listed in Table S1. *Gmt*, *Gaeumannomyces graminis* var. *tritici*. Numerical values at the bottom right of each bioassay represent the mean and standard deviation of the radius of the inhibition halo of three biological replicates. Bars, 5 mm.

We analysed the bioactivities of the wild‐type 9Rz4 and those of mutants defective in the biosynthesis of herbicolin A against 26 plant pathogenic fungi and oomycetes, using dual plate culture bioassays. We observed that herbicolin A was active against 68% of the plant pathogens tested, including *Helminthosporium sativum*, *Pyrenophora graminae*, *Cladosporium* sp., *Mycosphaerella graminícola*, *Phialophora fastigiata*, *Gaeumannomyces graminis* var. *tritici*, *Colletotrichum coccodes*, *Fusarium culmorum*, *Verticillium chlamydosporum*, *Botrytis allii*, *Botrytis cinerea*, *Botrytis fabae*, *Monilinia fructigena*, *Rhizoctonia solani*, *Rhizoctonia cerealis and Rhizoctonia tuliparum* (Figure 3)—fungi that include pathogens ranked in the top 10 in plant pathology worldwide (Dean et al., 2012). No bioactivities against *Pythium ultimum*, *Rhizoctonia oryzae*, *Armillaria mellea*, *Fusarium solani*, *Fusarium oxysporum*, *Penicillium crustosum* and *Chaetomium globosum* were detected (not shown). We also observed that herbicolin A was active against the ascomycete yeast *Schizosaccharomyces pombe* (Figure 3). Previous research showed that herbicolin A is also active against the human fungal pathogen *Candida albicans* (Winkelmann et al., 1980). Herbicolin A was recently shown to target ergosterol‐containing lipid rafts to disrupt fungal cell plasma membranes (Xu et al., 2022). Ergosterol is the most common sterol in fungal membranes (Rodrigues, 2018), which is in accordance with the broad spectrum of action of herbicolin A (Figure 3). In support of this data, oomycetes are sterol auxotrophs (Wang et al., 2021), which is probably the reason why we observed that herbicolin A is not active against *P. ultimum*.

**FIGURE 4:**
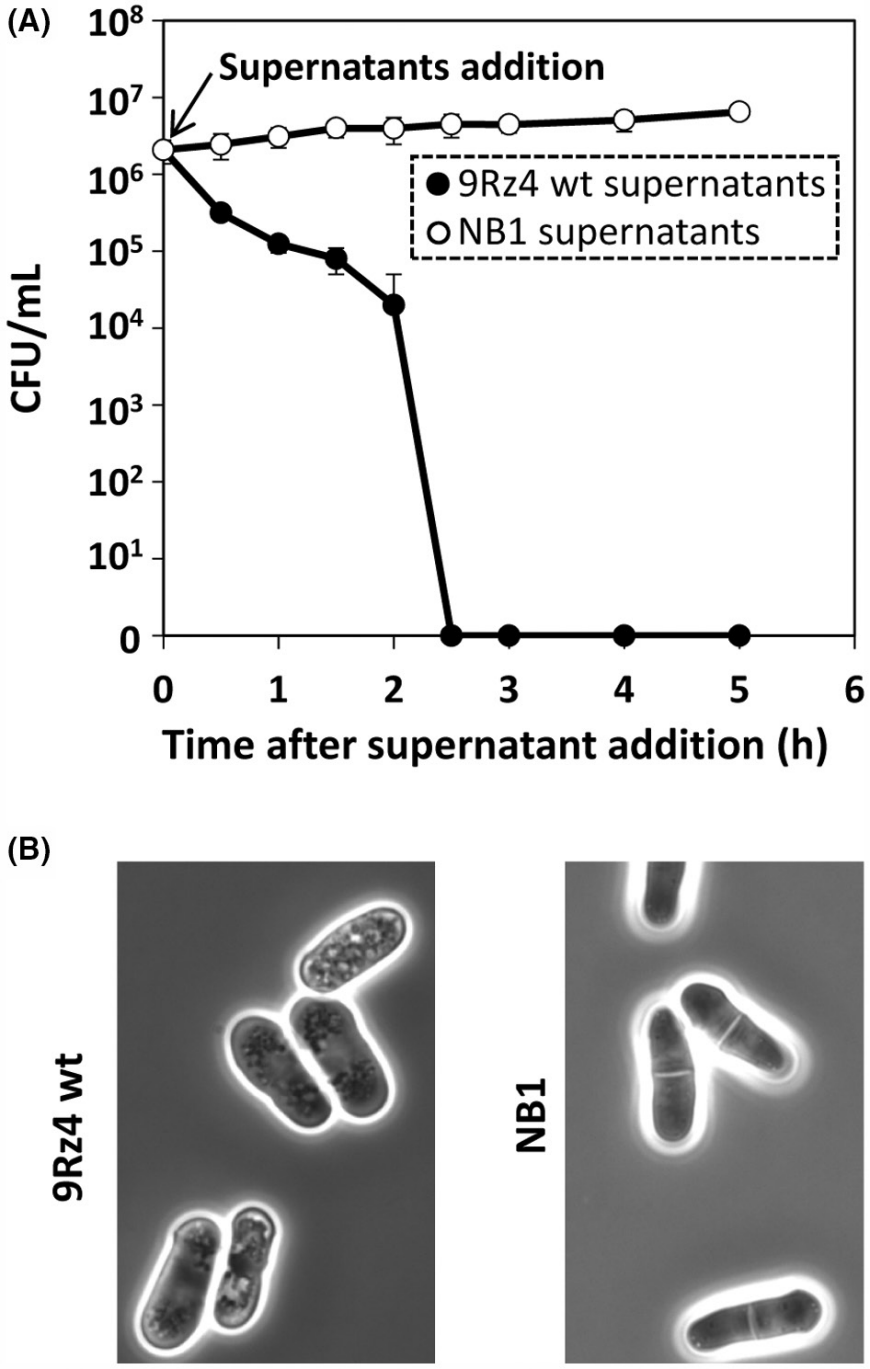
Impact of herbicolin A in the survival of *Schizosaccharomyces pombe*. (A) Survival of *S. pombe* after the addition of supernatants of *P. agglomerans* strains. Briefly, *S. pombe* cells were growth in YE medium (5 g/L yeast extract, 30 g/L glucose) at 32°C. At an OD_600_ of 0.25, *S. pombe* cells were pelleted by centrifugation and the pellet was resuspended in 5 ml of fresh 2× YE medium and 5 ml supernatants (1×, final concentration) of an overnight culture of 9Rz4 or NB1 grown in YE medium were added to the yeast cultures. Then, samples were taken every 30 min and the number of colony forming units (CFU) were determined. Data are the mean and standard deviation of three biological replicates. (B) Representative microscope images of *S. pombe* cells 2.5 h after the addition of 1× supernatants of 9RZ4 wild‐type and the mutant NB1. Microscope images were generated using an Olympus BX‐51 microscope with a 100× oil‐immersion lens. Images were acquired using a QICAM monochrome camera and QCapture Pro‐6 software.

To further investigate the antifungal activity of herbicolin A, supernatants from the wild‐type 9Rz4 and from an NB1 mutant, defective in herbicolin A production (Figure 2), were added to *S. pombe* cultures growing exponentially. A 10‐fold reduction in the number of viable *S. pombe* cells was observed 30 min after the addition of the wild‐type supernatants compared to the addition of the NB1 supernatants (Figure 4A). No live cells were detected 2.5 h after the addition of the wild‐type supernatants (Figure 4A). Microscopic inspection of these dead cells revealed different morphological changes, including irregular cell borders and the formation of stress granules (Figure 4B).

Previous data demonstrated that herbicolin A does not show toxic effects in plant leaves and fruits, and a herbicolin A producing bacterium was shown to protect plants against fungal pathogens (Xu et al., 2022). Multiple *P. agglomerans* strains were shown to protect plants against a wide range of phytopathogens and some *P. agglomerans* isolates are commercially available as biopesticides (Dutkiewicz et al., 2016; Lahlali et al., 2020; Xu et al., 2022). To rule out any negative effect of 9Rz4 on plant growth, we conducted maize root colonization assays and compare the fitness of inoculated and non‐inoculated maize plants 10 days after the inoculation. No statistically significant differences in aerial part size or root weight were observed between treatments (Figure S2A,B), laying the first foundations for the safe use of 9Rz4 as a rhizosphere biocontrol agent. Subsequently, to evaluate the implications of herbicolin A production in plant root colonization, additional root colonization assays were conducted with the wild‐type 9Rz4 and the herbicolin A deficient strain NB1. We found that both strains colonize the rhizosphere of maize with identical density, ~10^8^ bacteria per gram of root (Figure S2C), as an indication that the biosynthesis of the antifungal metabolite does not affect the fitness of 9Rz4 in the rhizosphere.

### Plasmid carriage does not affect antifungal production

The analysis of the genome of 9Rz4 associated the yellow pigmentation of this strain with the production of carotenoids since the biosynthetic cluster responsible for the production of these pigments was located in the p9Rz4_1 plasmid. Carotenoid production has been described in other strains of the *Pantoea* genus, which often have the corresponding biosynthetic cluster on a plasmid (Choi et al., 2021; Smits et al., 2010). The biosynthesis of secondary metabolites is energy demanding (Krell & Matilla, 2022; Liu et al., 2013) and plasmid carriage can pose an energy cost to the bacterial host (Trautwein et al., 2016; Vial & Hommais, 2020). Indeed, some studies have reported an effect of plasmid load on the biosynthesis of secondary metabolites (Thomas et al., 1991). To investigate the role of plasmid carriage in herbicolin A production, a plasmid curation protocol described previously (Smits et al., 2010) was used to generate a non‐pigmented variant of 9Rz4, strain 9Rz4‐W (Figure S3). This lack of pigmentation was attributed by PCR screening to the loss of the p9Rz4_1 plasmid containing the carotenoid biosynthetic gene cluster of 9Rz4. This strain 9Rz4‐W showed the same antifungal properties against *V. dahliae* as those observed in the wild‐type strain (Figure S3), suggesting that no regulatory elements are present in p9Rz4_1 that modulate herbicolin A production.

### Modulation of herbicolin A production by quorum‐sensing

**FIGURE 5:**
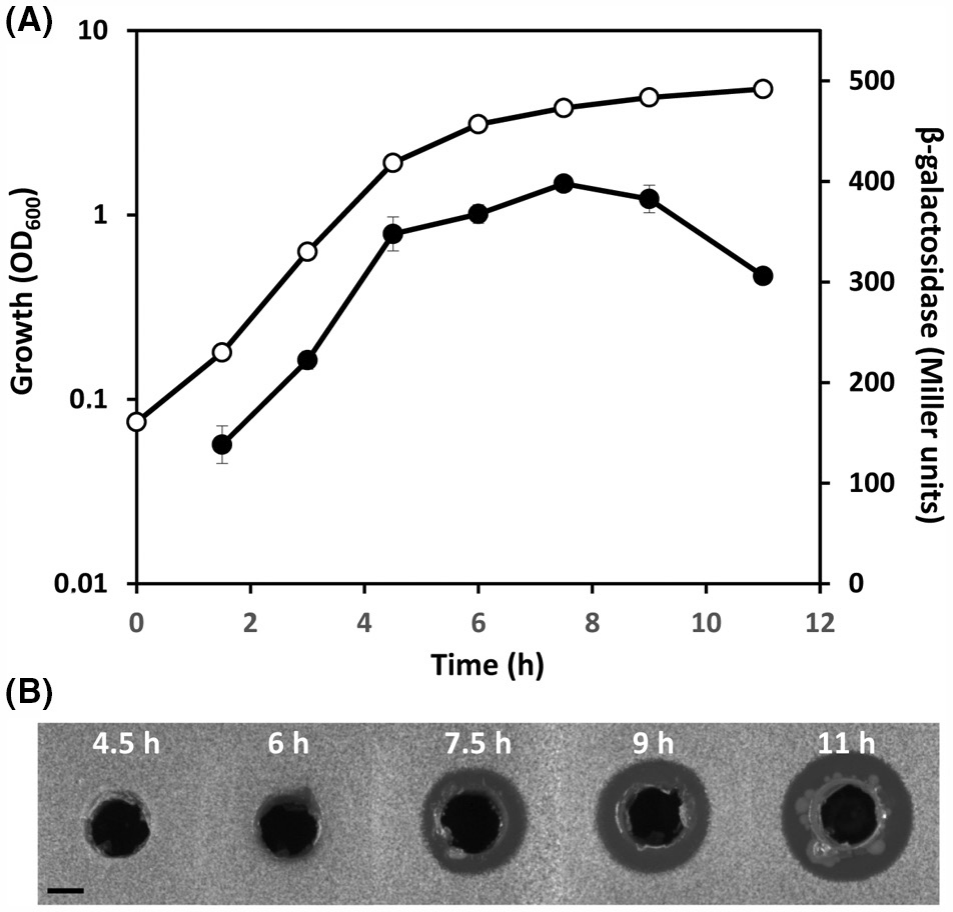
Expression of the herbicolin A biosynthetic cluster throughout the growth phase in *Pantoea agglomerans* 9Rz4. (A) β‐galactosidase activity (filled symbols) throughout growth (open symbols) measured from a chromosomal fusion *hbcB*::*lacZ* (strain NB1) in *P. agglomerans* 9Rz4 in LB medium at 25°C. Data are the mean and standard deviation of three biological replicates. β‐galactosidase activity was measured as described previously (Miller, 1972) using 2‐Nitrophenyl‐β‐d‐galactopyranoside (Sigma‐Aldrich; Cat No. N1127) as substrate. (B) Herbicolin A production by *P. agglomerans* 9Rz4 throughout growth in LB medium at 25°C. For the bioassays, a *Schizosaccharomyces pombe* top agar lawn was prepared and 300 μl of filter‐sterilized supernatants of 9Rz4 was added to holes punched in the *S. pombe* bioassay plates. The bioassays were repeated three times and representative pictures are shown. Pictures were taken after 48 h of incubation at 30°C. The size of the inhibition halos is indicative of the susceptibility of *S. pombe* to herbicolin A. Bar, 5 mm.

To investigate the expression of the herbicolin A gene cluster, we used a chromosomal transcriptional fusion located in the *hbcB* gene (strain NB1). Our β‐galactosidase assays revealed that the transcription of the gene cluster starts at early‐exponential phase of growth, with maximum expression at early‐stationary phase of growth (Figure 5A). The analysis of herbicolin A production throughout the growth curve showed that the expression of the biosynthetic cluster correlates perfectly with the presence of the antifungal antibiotic in cell‐free supernatants (Figure 5B).

**FIGURE 6:**
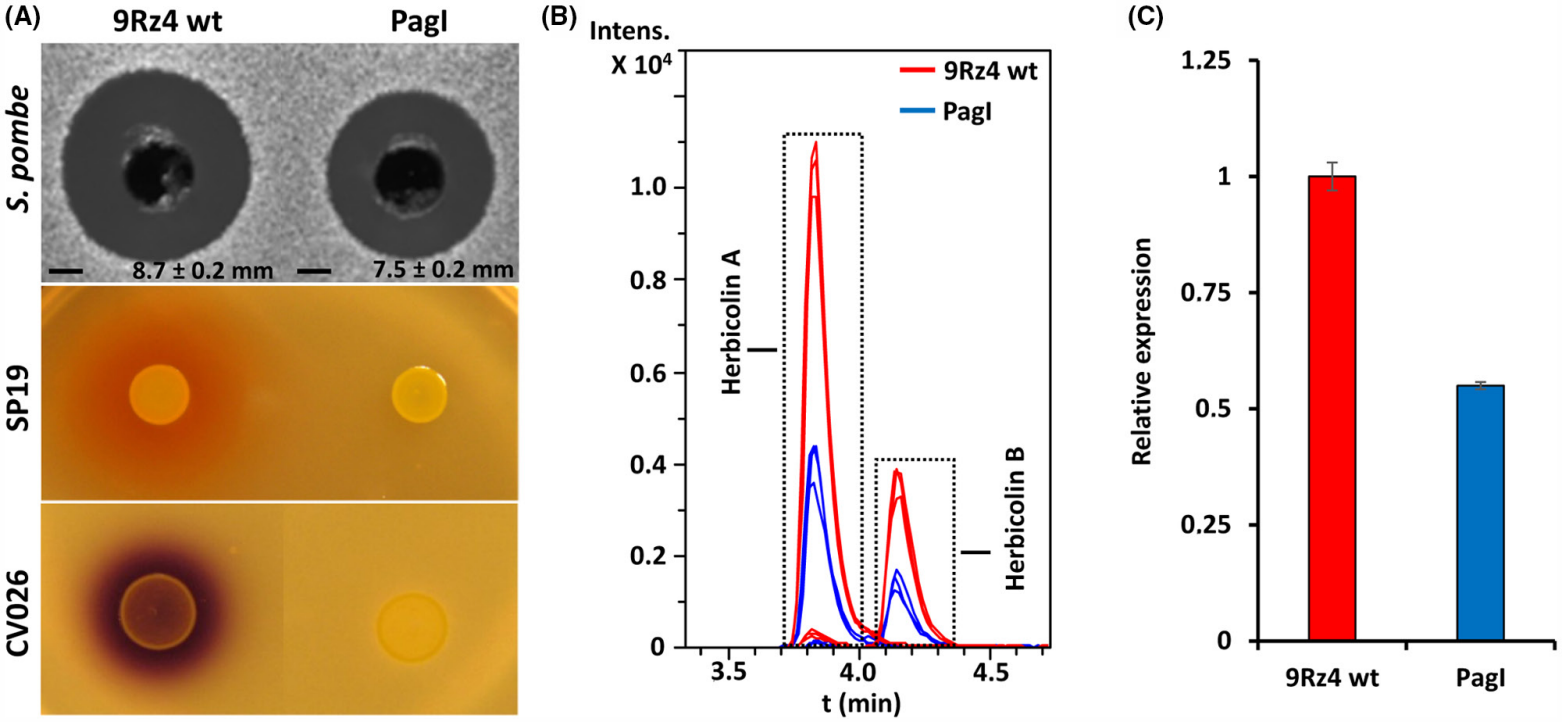
Effect of quorum sensing on herbicolins A and B biosynthesis in *P. agglomerans* 9Rz4. (A) Bioctivity and acyl‐homoserine lactone (AHL) production in *P. agglomerans* 9Rz4 strains. Shown are the growth inhibitory capacities of culture supernatants of the 9Rz4 strains to *Schizosaccharomyces pombe*. Prodigiosin and violacein production by the biosensor strains *Serratia* SP19 and *Chromobacterium violaceum* CV026, respectively, in response to AHLs produced by the 9Rz4 strains is also shown. PagI is a mutant strain defective in the *pagI* gene encoding the AHL synthase of the quorum sensing system PagIR. For the antifungal bioassays, a *S. pombe* top agar lawn was prepared and 300 μl of overnight culture filter‐sterilized supernatants of 9Rz4 strains grown in LB was added to holes punched in the *S. pombe* bioassay plates. For the SP19 and CV026 assays, 5 μl of an overnight culture of the selected strains was spotted on the surface of the bioassay plates. The bioassays were repeated at least three times and representative results are shown. In all cases, pictures were taken after 48 h of incubation at 30°C (*S. pombe*) or 25°C (SP19 and CV026). (B) LC‐HRMS analysis of the supernatants of 9Rz4 strains. Shown are extracted ion chromatograms (EIC) of three biological replicates corresponding to an *m/z* of 659.385 ± 0.005 (theoretical value for [M + 2H]^2+^ in herbicolin A) and to an *m*/*z* of 569.845 ± 0.005 (theoretical value for [M + 2H]^2+^ in herbicolin B). Relative abundance of herbicolins A and B in the supernatants of the *P. agglomerans* 9Rz4 strains is shown in Figure S4 and it was done through the measurement of peak areas in the EICs. (C) Impact of quorum sensing on *hbcA* transcript levels measured by quantitative real‐time PCR (qPCR). Bacteria were grown in LB medium at 25°C and samples for RNA isolation were taken at early stationary phase of growth (OD_600_ ~ 3.5), when the expression of the herbicolin A gene cluster was maximal (see Figure 5A). The values showed the average expression relative to the wild‐type expression. Data are the mean and standard deviations from three biological replicates. RNA extraction, cDNA synthesis and quantitative real‐time PCR analyses were conducted as described previously (Rico‐Jiménez et al., 2022). Oligonucleotides used are detailed in Table S1. The relative gene expression was calculated using the critical threshold (ΔΔ*C*_t_) method (Pfaffl, 2001) and using the *gyrB* gene as the internal control to normalize the data.

The expression pattern of the herbicolin A gene cluster suggested a possible role of quorum sensing (QS) in regulation of antifungal antibiotic production. Analysis of the genome sequence of *P. agglomerans* 9Rz4 revealed the presence of a single QS locus, PagIR, whose acyl‐homoserine lactone (AHL) synthase, PagI, has been previously associated with the production of N‐butanoyl‐L‐homoserine lactone (C4‐HSL) and N‐hexanoyl‐L‐homoserine lactone (C6‐HSL) as the major and minor QS signals, respectively (Chalupowicz et al., 2008). Given that QS was shown to regulate the production of multiple antibiotics in enterobacteria, including oocydin A (Matilla et al., 2015), prodigiosin (Williamson et al., 2006), a carbapenem (Coulthurst et al., 2005) and solanimycin (Matilla et al., 2022), we examined its possible role in the modulation of herbicolin A biosynthesis in 9Rz4. To this end, we screened a random transposon mutant library of 9Rz4 scoring for mutants defective in AHL production, using the *Serratia* SP19 biosensor strain (Poulter et al., 2010). A *pagI* mutant, defective in the production of AHLs (Figure 6A), was isolated and its phenotypic characterization revealed a slight reduction in the bioactivity against *S. pombe* with filter‐sterilized supernatants of the *pagI* mutant relative to the wild‐type strain (Figure 6A). Subsequent LC‐HRMS measurements of the supernatants revealed a 2.5 ± 0.4 and 2.6 ± 0.4 fold reduction in the herbicolins A and B levels, respectively, in the *pagI* mutant strain compared to the parental strain (Figure 6B and Figure S4). The production of herbicolins A and B in the *pagI* mutant was fully restored by the addition of supernatants from an NB1 mutant (e.g. acyl‐homoserine lactone producer; herbicolin A deficient) but not when supernatants of the *pagI* mutant were added; as shown in the antibiosis assays against *S. pombe* and in the LC‐HRMS measurements of herbicolins A and B levels (Figure S5). The addition of 2 μM C4‐HSL or C6‐HSL to bacterial cultures of the wild‐type 9Rz4 or the *pagI* mutant did not result precocious production of the antifungal molecule (Evans, 2010), implying a complex regulatory network that modulates herbicolin A production. Quantitative real‐time PCR analyses were conducted to measure transcript levels of *hbcA* in the wild‐type 9Rz4 and its *pagI* mutant strain, which revealed that the transcriptional levels of the herbicolin A gene cluster were reduced by ~45% in a *pagI* deficient background (Figure 6C).

## CONCLUSIONS AND FUTURE DIRECTIONS

Fungal and oomycete crop pathogens represent an alarming problem in agriculture associated with a global food security risk. Indeed, they cause crop losses that can amount to up to 80% under appropriate conditions (Fones et al., 2020). This is aggravated by climate change, monoculture practices and the loss of crop diversity. In addition, the widespread use of single target synthetic fungicides encourages the development of resistance to pesticides (Fones et al., 2020). Although biopesticides currently account for only ~6% of the global pesticide market, the annual growth rate in biopesticide sales is estimated at 10%–20%, encouraged by traits such as their low rate of resistance evolution, their high performance and their biosafety and biodegradability (Marrone, 2019).

Actinobacteria currently represent the main source of antibiotics for clinical and agricultural uses (Krell & Matilla, 2022). However, as this study has confirmed, plant‐associated bacteria represent a potential source for novel antibiotics (Gavriilidou et al., 2022; Matilla et al., 2022; Mohite et al., 2022; Roca & Matilla, 2022). Remarkably, the biosynthesis of specific antibiotic compounds by plant‐associated bacteria has been shown to play a key role in the protection of agricultural crops by beneficial microbes (Carrión et al., 2019; Hou & Kolodkin‐Gal, 2020; Mendes et al., 2011). Taken together, plant‐associated microorganisms have a very promising potential to provide solutions for existing agricultural challenges. Hence, future directions are now aimed at engineering plant microbiomes to promote beneficial interactions to improve plant productivity and health (Ke et al., 2021).

## AUTHOR CONTRIBUTIONS

**Miguel A. Matilla:** Conceptualization (lead); data curation (lead); formal analysis (lead); funding acquisition (lead); investigation (lead); methodology (lead); project administration (lead); resources (lead); supervision (lead); validation (lead); visualization (lead); writing – original draft (lead); writing – review and editing (lead). **Terry J. Evans:** Formal analysis (equal); investigation (equal); methodology (equal); writing – review and editing (equal). **Jesús Martín:** Data curation (equal); formal analysis (equal); investigation (equal); methodology (equal); software (equal); validation (equal); writing – review and editing (equal). **Zulema Udaondo:** Data curation (equal); investigation (equal); methodology (equal); software (equal); validation (equal); writing – review and editing (equal). **Cristina Lomas‐Martínez:** Investigation (equal); methodology (equal); writing – review and editing (equal). **Míriam Rico‐Jiménez:** Investigation (equal); methodology (equal); writing – review and editing (equal). **Fernando Reyes:** Data curation (equal); formal analysis (equal); investigation (equal); methodology (equal); software (equal); validation (equal); writing – review and editing (equal). **George P. C. Salmond:** Conceptualization (lead); formal analysis (lead); funding acquisition (lead); investigation (lead); methodology (lead); project administration (lead); resources (lead); supervision (lead); writing – original draft (lead); writing – review and editing (lead).

## FUNDING INFORMATION

Work in the Salmond laboratory was supported by the Biotechnology and Biological Sciences Research Council (BBSRC; UK) through award BB/N008081/1 to GPCS. Work in the Matilla laboratory was supported by a grant from the Spanish Ministry for Science and Innovation/*Agencia Estatal de Investigación* https://doi.org/10.13039/501100011033 (PID2019‐103972GA‐I00).

## CONFLICT OF INTEREST

The authors declare that there is no conflict of interest.

## Supporting information


Appendix S1

## Data Availability

The genome of *Pantoea agglomerans* 9RZ4 has been deposited in DDBJ/EMBL/GenBank under the GenBank accession number JANHBZ000000000.
