# Supplementary material for: Herbicolin A production and its modulation by quorum sensing in a Pantoea agglomerans rhizobacterium bioactive against a broad spectrum of plant‐pathogenic fungi
Source: Microb Biotechnol. 2022 Dec 18;16(8):1690–700. doi: 10.1111/1751-7915.14193 (PMC10364316; doi:10.1111/1751-7915.14193)
Supplement: Supplementary file 1 — Appendix S1 [file MBT2-16-1690-s001.docx]

**SUPPLEMENTARY MATERIAL TO:**

**Herbicolin A production and its modulation by quorum sensing in a *Pantoea agglomerans* rhizobacterium bioactive against a broad spectrum of plant-pathogenic fungi**

**Miguel A. Matilla^1,2^*, Terry J. Evans^2^, Jesús Martín^3^, Zulema Udaondo^4^, Cristina Lomas-Martínez^1^, Míriam Rico-Jiménez^1^,** **Fernando Reyes^3^, George P.C. Salmond^2^***

^1^Department of Biotechnology and Environmental Protection, Estación Experimental del Zaidín, Consejo Superior de Investigaciones Científicas, Prof. Albareda 1, Granada 18008, Spain.

^2^Department of Biochemistry, University of Cambridge, Tennis Court Road, Cambridge, CB2 1QW, United Kingdom.

^3^Fundación MEDINA, Centro de Excelencia en Investigación de Medicamentos Innovadores en Andalucía, Avda del Conocimiento 34, Armilla, 18016 Granada, Spain.

^4^Department of Biomedical Informatics, University of Arkansas for Medical Sciences, Little Rock, AR 72205, USA.

*Address correspondence to Miguel A. Matilla, [miguel.matilla@eez.csic.es](mailto:miguel.matilla@eez.csic.es), Tel. +34 958 526506; Fax +34 958 181609; George P.C. Salmond, [gpcs2@cam.ac.uk](mailto:gpcs2@cam.ac.uk), Tel: +44 (0)1223 333650; Fax: +44 (0)1223 766108.

**ORCID numbers Miguel A. Matilla:** **0000-0002-8468-9604**

**Terry J. Evans: 0000-0001-8976-5197**

**Zulema Udaondo:** [**0000-0003-3445-6842**](https://www.scopus.com/redirect.uri?url=https://orcid.org/0000-0003-3445-6842&authorId=55246829500&origin=AuthorProfile&orcId=0000-0003-3445-6842&category=orcidLink)

**Fernando Reyes: 0000-0003-1607-5106**

**George P.C. Salmond: 0000-0002-5197-2198**

**Supplementary Table S1. Bacteria, oomycete, fungi, phages, plasmids and oligonucleotides used in this study.**

| **Bacterial strains** | | **Genotype or relevant characteristic^a^** | | **Reference or source** | | |
| --- | --- | --- | --- | --- | --- | --- |
| *Escherichia coli* β2163 | | F^-^ RP4-2-Tc::Mu Δ*dapA*::(*erm-pir*); Km^R^ Em^R^ | | (Demarre *et al.*, 2005) | | |
| *Pantoea agglomerans* 9Rz4 | | Oilseed rape isolate. Wild type. Natural lac- strain. | | (Berg *et al.*, 2002) | | |
| *P. agglomerans* NB1 | | 9Rz4 transposon mutant *hcbB*::Tn-KRCPN1; Km^R^. *hcbB* chromosomal *lacZ* transcriptional fusion. | | This study | | |
| *P. agglomerans* NB7 | | 9Rz4 transposon mutant *hcbE*::Tn-KRCPN1; Km^R^ | | This study | | |
| *P. agglomerans* NB9 | | 9Rz4 transposon mutant *hcbD*::Tn-KRCPN1; Km^R^ | | This study | | |
| *P. agglomerans* NB13 | | 9Rz4 transposon mutant *hcbH*::Tn-KRCPN1; Km^R^ | | This study | | |
| *P. agglomerans* NB14 | | 9Rz4 transposon mutant *hcbE*::Tn-KRCPN1; Km^R^ | | This study | | |
| *P. agglomerans* NB15 | | 9Rz4 transposon mutant *hcbI*::Tn-KRCPN1; Km^R^ | | This study | | |
| *P. agglomerans* NB18 | | 9Rz4 transposon mutant *hcbE*::Tn-KRCPN1; Km^R^ | | This study | | |
| *P. agglomerans* PagI | | 9Rz4 transposon mutant *pagI*::Tn-Km; Km^R^ | | This study | | |
| *P. agglomerans* 9Rz4-W | | 9Rz4 variant curated of plasmid p9Rz4_1 | | This study | | |
| *Serratia* SP19 | | *smaI*::mini-Tn*5*-Sm/Sp, *pigX*::Tn-DS1028, *pigZ*::mini-Tn5-*lacZ*1; AHLs bioassay strain; Sp^R^, Cm^R^, Km^R^ | | (Poulter *et al.*, 2010) | | |
| *Chromobacterium violaceum* CV026 | | *cviI*::Tn*5*, AHLs bioassay strain | | (McClean *et al.*, 1997) | | |
| **Fungi/oomycete strains** | | **Genotype or relevant characteristic** | | **Reference or source** | | |
| *Schizosaccharomyces pombe* | | Wild type | | J. Mata | | |
| *Pythium ultimum* | | Wild type, plant pathogen. Isolated from diseased plants – Department of Plant Sciences, Cambridge (UK) | | C.A. Gilligan | | |
| *Verticillium dahliae* 5368 | | Wild type, plant pathogen. Isolated from tomato diseased plants. | | R. Cooper | | |
| *Helminthosporium sativum* | | Wild type, plant pathogen. Isolated from diseased plants – Department of Plant Sciences, Cambridge (UK) | | C.A. Gilligan | | |
| *Pyrenophora graminae* | | Wild type, plant pathogen. Isolated from diseased plants – Department of Plant Sciences, Cambridge (UK) | | C.A. Gilligan | | |
| *Cladosporium* sp. C45 | | Wild type, plant pathogen. Isolated from diseased plants – Department of Plant Sciences, Cambridge (UK) | | C.A. Gilligan | | |
| *Mycosphaerella graminicola* | | Wild type, plant pathogen. Isolated from diseased plants – Department of Plant Sciences, Cambridge (UK) | | C.A. Gilligan | | |
| *Penicillium crustosum* | | Wild type, plant pathogen. Isolated from diseased plants – Department of Plant Sciences, Cambridge (UK) | | C.A. Gilligan | | |
| *Phialophora fastigiata* | | Wild type, plant pathogen. Isolated from diseased plants – Department of Plant Sciences, Cambridge (UK) | | C.A. Gilligan | | |
| *Gaeumannomyces graminis* var. *tritici* (*Gmt*) | | Wild type, plant pathogen. Isolated from diseased plants – Department of Plant Sciences, Cambridge (UK) | | C.A. Gilligan | | |
| *Colletotrichum coccodes* | | Wild type, plant pathogen. Isolated from diseased plants – Department of Plant Sciences, Cambridge (UK) | | C.A. Gilligan | | |
| *Fusarium culmorum* | | Wild type, plant pathogen. Isolated from diseased plants – Department of Plant Sciences, Cambridge (UK) | | C.A. Gilligan | | |
| *Fusarium oxysporum* | | Wild type, plant pathogen. Isolated from diseased plants – Department of Plant Sciences, Cambridge (UK) | | C.A. Gilligan | | |
| *Fusarium solani* | | Wild type, plant pathogen. Isolated from diseased plants – Department of Plant Sciences, Cambridge (UK) | | C.A. Gilligan | | |
| *Chaetomium globosum* | | Wild type, plant pathogen. Isolated from diseased plants – Department of Plant Sciences, Cambridge (UK) | | C.A. Gilligan | | |
| *Verticillium cinnabarinum* | | Wild type, plant pathogen. Isolated from diseased plants – Department of Plant Sciences, Cambridge (UK) | | C.A. Gilligan | | |
| *Verticillium chlamydosporum* | | Wild type, plant pathogen. Isolated from diseased plants – Department of Plant Sciences, Cambridge (UK) | | C.A. Gilligan | | |
| *Botrytis allii* | | Wild type, plant pathogen. Isolated from diseased plants – Department of Plant Sciences, Cambridge (UK) | | C.A. Gilligan | | |
| *Botrytis cinerea* | | Wild type, plant pathogen. Isolated from diseased plants – Department of Plant Sciences, Cambridge (UK) | | C.A. Gilligan | | |
| *Botrytis fabae* | | Wild type, plant pathogen. Isolated from diseased plants – Department of Plant Sciences, Cambridge (UK) | | C.A. Gilligan | | |
| *Monilinia fructigena* | | Wild type, plant pathogen. Isolated from diseased plants – Department of Plant Sciences, Cambridge (UK) | | C.A. Gilligan | | |
| *Armillaria mellea* | | Wild type, plant pathogen. Isolated from diseased plants – Department of Plant Sciences, Cambridge (UK) | | C.A. Gilligan | | |
| *Corticium solani* | | Wild type, plant pathogen. Isolated from diseased plants – Department of Plant Sciences, Cambridge (UK) | | C.A. Gilligan | | |
| *Rhizoctonia solani* | | Wild type, plant pathogen. Isolated from diseased plants – Department of Plant Sciences, Cambridge (UK) | | C.A. Gilligan | | |
| *Rhizoctonia cerealis* | | Wild type, plant pathogen. Isolated from diseased plants – Department of Plant Sciences, Cambridge (UK) | | C.A. Gilligan | | |
| *Rhizoctonia tuliparum* | | Wild type, plant pathogen. Isolated from diseased plants – Department of Plant Sciences, Cambridge (UK) | | C.A. Gilligan | | |
| *Rhizoctonia oryzae* | | Wild type, plant pathogen. Isolated from diseased plants – Department of Plant Sciences, Cambridge (UK) | | C.A. Gilligan | | |
| **Phages** | | | | | | |
| ϕOT8 | | Generalized transducing phage for *Pantoea agglomerans* 9Rz4 | | (Evans *et al.*, 2010) | | |
| **Plasmids** | | **Relevant characteristic^a^** | | **Source** | | |
| pKRCPN1 | | Km^R^, Tc^R^; Derivative of pDS1028*uidA* with the *uidA* and *cat* genes replaced with *lacZ* and *km* genes. | | (Monson *et al.*, 2015) | | |
| pNRW124 | | Km^R^; Plasmid for transposon mutagenesis. This plasmid was constructed by digesting pKRCPN1 with KpnI to remove the *lacZ* gene and religating. | | Salmond lab. Unpublished | | |
| **Oligonucleotides** | | | | | |  |
| **Name** | **Sequence (5´- 3')** | | **Description** | | **Source** |  |
| MAMV1 | GGAATTGATCCGGTGGATG | | Tn-KRCPN1 specific primer | | (Matilla *et al.*, 2012) |  |
| MAMV2 | GCATAAAGCTTGCTCAATCAATCAC | | Tn-KRCPN1 specific primer | | (Matilla *et al.*, 2012) |  |
| MAMV3 | CTAAGCTGATCCGGTGGATG | | mini-Tn*5-*Sm/Sp specific primer | | (Matilla *et al.*, 2014) |  |
| MAMV4 | AACGGTTTACAAGCATAAAGC | | mini-Tn*5-*Sm/Sp specific primer | | (Matilla *et al.*, 2014) |  |
| PF106 | GACCACACGTCGACTAGTGCNNNNNNNNNNAGAG | | Random primed PCR primer 1 | | (Fineran *et al.*, 2005) |  |
| PF107 | GACCACACGTCGACTAGTGCNNNNNNNNNNACGCC | | Random primed PCR primer 2 | | (Fineran *et al.*, 2005) |  |
| PF108 | GACCACACGTCGACTAGTGCNNNNNNNNNNGATAC | | Random primed PCR primer 3 | | (Fineran *et al.*, 2005) |  |
| PF109 | GACCACACGTCGACTAGTGC | | Random primed PCR adapter primer | | (Fineran *et al.*, 2005) |  |
| CRT-F | GTGCCGTTTCTGTCATTCCG | | Amplify a carotenoid biosynthesis gene in plasmid p9Rz4_1 | | This study |  |
| CRT-R | CATGTAATGCTGTTCCAGATAGTCG | | Amplify a carotenoid biosynthesis gene in plasmid p9Rz4_1 | | This study |  |
| gyrB_9Rz4-F | CCGTGAAGGTCTGATCGCTG | | Forward primer for quantitative real-time PCR | | This study |  |
| gyrB_9Rz4-R | GGTATTCAGCCAGCAGTTCATTC | | Reverse primer for quantitative real-time PCR | | This study |  |
| hbcA-qPCR-F | CAACACCTGGCGGCTTATTAC | | Forward primer for quantitative real-time PCR | | This study |  |
| hbcA-qPCR-R | CGCTTGCCAGTAGGAATGATC | | Reverse primer for quantitative real-time PCR | | This study |  |

### ^a^Ap, ampicillin; Cm, Chloramphenicol; Em, *e*rythromycin; Km, kanamycin; Sm, streptomycin; Sp, spectinomycin; Tc, tetracycline.

### **Supplementary Figures**


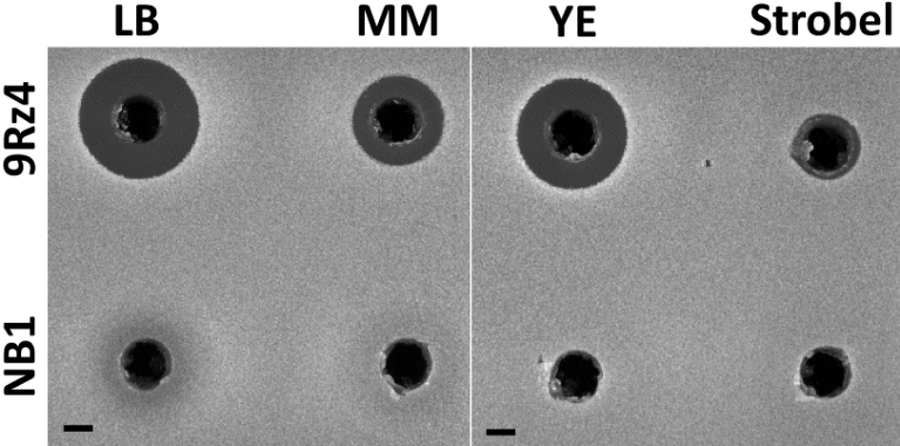


**Supplementary Figure S1. Halos of antibiosis of filter-sterilized supernatants of *Pantoea agglomerans* 9Rz4 grown in different culture media.** Growth inhibition of the ascomycete yeast *Schizosaccharomyces pombe* (herbicolin A sensitive) with culture supernatants of *P. agglomerans* 9Rz4 and the herbicolin A deficient mutant NB1 grown overnight in LB, minimal medium (MM), YE and Strobel medium (Strobel *et al.*, 1999) at 25 ºC. For the bioassays, a *S.* *pombe* top agar lawn was prepared in YE-agar and 300 µl of filter-sterilized supernatants were added to holes punched in the *S. pombe* bioassay plates. The size of the inhibition halos is indicative of the susceptibility of *S. pombe* to herbicolin A. The bioassays were repeated three times and representative pictures are shown. Picture were taken after 48 h of incubation at 30 ºC. The radius of the halos from three biological replicas is 7.0 ± 0.2 mm (LB), 4.1 ± 0.1 (MM), 6 ± 0.1 mm (YE) and 1.8 ± 0.05 mm (Strobel medium). Bars, 5 mm.

**
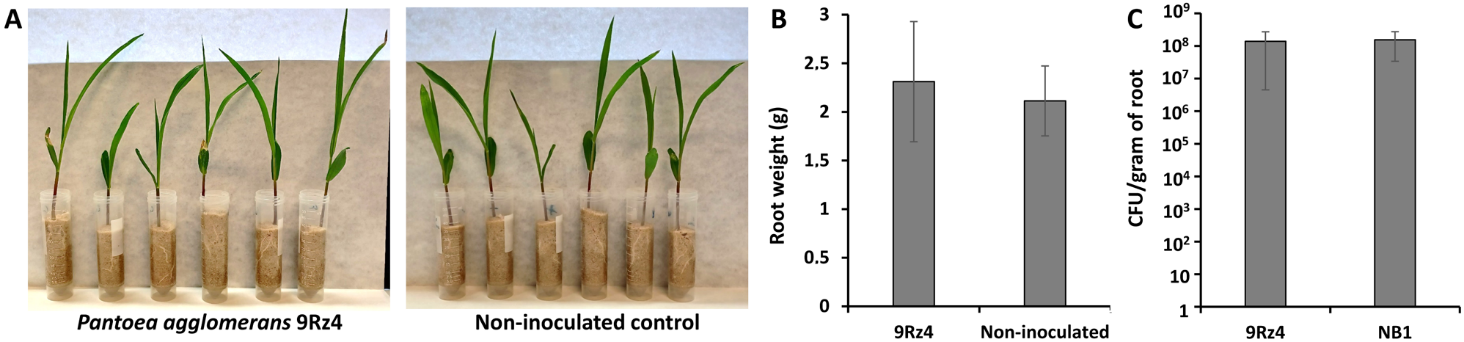
**

**Supplementary Figure S2. *Pantoea agglomerans* 9Rz4 maize root colonization and its effect on plant growth. A**, Maize plants 10 days after inoculation with 9RZ4. Non-inoculated plants were included as control. **B**, Root weight of maize plants shown in Fig. S2A. Shown are mean and standard deviation of six different plants. No statistically significant differences in root weight were observed between inoculated and non-inoculated plants. **C**, Maize root colonization assays of *P. agglomerans* 9Rz4 and its mutant strain NB1. Shown are mean and standard deviation of six different plants. In A-C, sterilization, germination and inoculation of maize seeds was carried out as described previously (Matilla *et al.*, 2007), with minor modifications. Briefly, sterile maize seeds were incubated for 45 min at 30 ºC with a 10^7^ CFU/mL of *Pantoea agglomerans* 9Rz4 strains. Thereafter, seeds were rinsed with sterile deionized water and planted in 50 mL tubes containing 40 g of sterile washed silica sand and 10% (v/w) plant nutrient solution supplemented with Fe-EDTA and micronutrients, as described previously (Matilla *et al.*, 2007). Plants were maintained at 24 ºC with a daily light period of 16 h for 10 days.

**
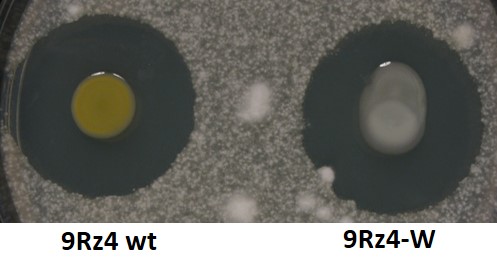
**

**Supplementary Figure S3. Effect of plasmid carriage on the antifungal properties of *P. agglomerans* 9Rz4.** Bioactivity against *Verticillium dahlia*e of 9Rz4 and a 9Rz4 variant (9Rz4-W) lacking plasmid p9Rz4_1. Pictures were taken after 96 h of incubation at 25 °C.


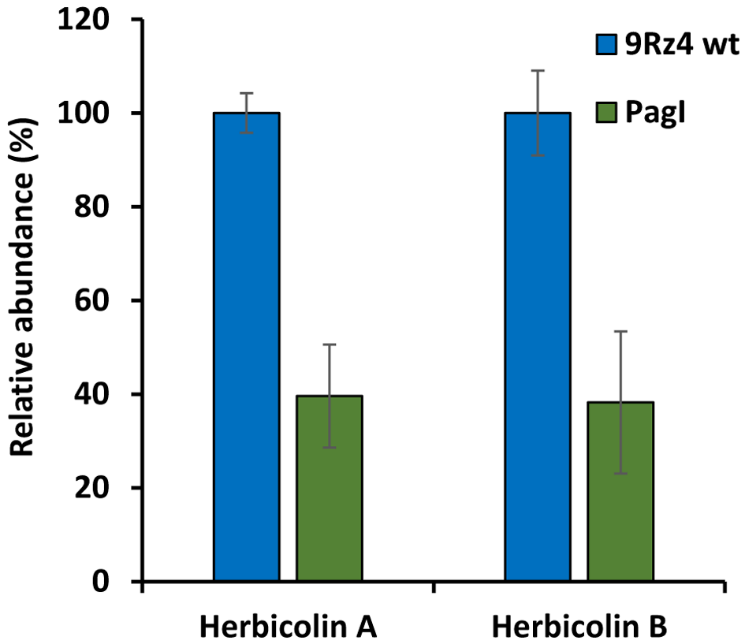


**Supplementary Figure S4. Herbicolins A and B production is reduced in the quorum sensing mutant defective in the acyl-homoserine lactone synthase PagI.** Abundance of herbicolins A and B relative to the wild type 9Rz4 in the supernatants of the *P. agglomerans* 9Rz4 strains. Data are the mean and standard deviations from three biological replicates and correspond to the intensity (area under the peak) of “extracted ion chromatograms” (EIC) shown in Fig. 6B. Note that the areas derived from the EICs are not comparable between compounds (e.g. herbicolin A vs herbicolin B) as these areas depend on the ionization efficiency of each compound. As shown in Fig. 1A, herbicolin B is found at trace levels in the 9Rz4 supernatants based on LC-HRMS analyses.


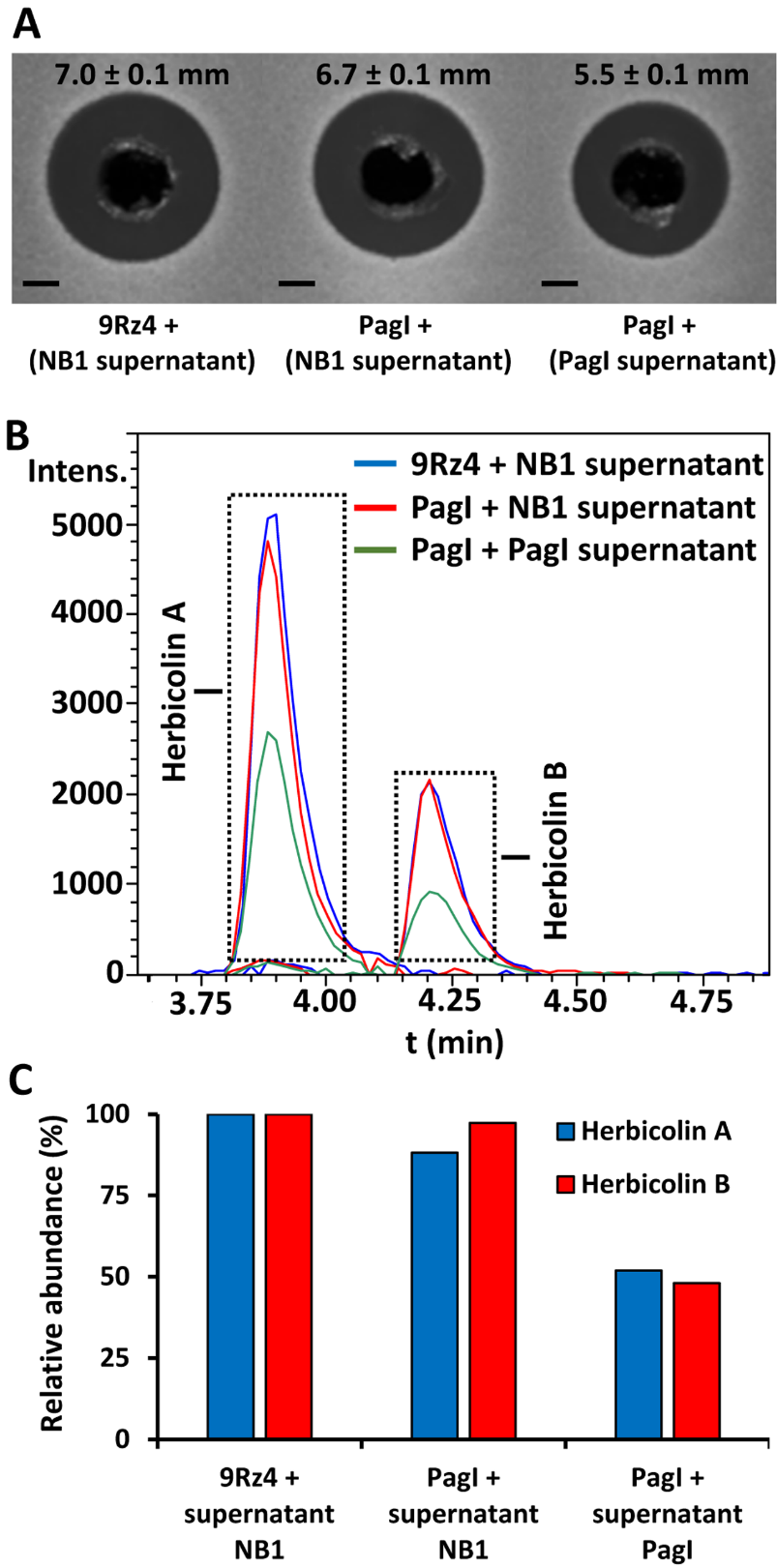


**Supplementary Figure S5: Complementation of herbicolins A and B production in the quorum sensing mutant defective in the acyl-homoserine lactone synthase PagI.** **A**, Growth inhibition of *Schizosaccharomyces* *pombe* with culture supernatants of *Pantoea agglomerans* strains. For the assays, *P. agglomerans* strains were inoculated at an initial OD_660_ of 0.05 in 10 mL of LB medium containing 1 mL supernatants of overnight cultures of *P. agglomerans* NB1 (herbicolin A defective; wild type in acyl-homoserine lactone production) or *P. agglomerans* PagI (defective in the synthesis of acyl-homoserine lactones) grown in LB medium. Then, bacterial cultures were grown overnight at 25 ºC, at which time the supernatants were collected, filter-sterilized and characterized chemically and biologically. For the bioassays, a *Schizosaccharomyces* *pombe* top agar lawn was prepared and 300 µL of filter-sterilized supernatants were added to holes punched in the *S. pombe* bioassay plates. Pictures were taken after 48 h of incubation at 30 ºC. Numerical values indicate the mean and standard deviation of the radius of the inhibition halo of three biological replicates. Bars, 5 mm. **B**, Extracted ion chromatograms (EIC) corresponding to an *m/z* of 659.385 ± 0.005 (theoretical value for [M+2H]^2+^ in herbicolin A) and to an *m/z* of 569.845 ± 0.005 (theoretical value for [M+2H]^2+^ in herbicolin B). **C**, Abundance of herbicolins A and B relative to the wild type strain 9Rz4 in the supernatants of the *P. agglomerans* 9Rz4 strains through the measurement of peak areas in the EICs shown in Fig. S5B. In B and C, note that the areas derived from the EICs are not comparable between compounds (e.g. herbicolin A vs herbicolin B) as these areas depend on the ionization efficiency of each compound. As shown in Fig. 1A, herbicolin B is found at trace levels in the 9Rz4 supernatants based on LC-HRMS analyses.

### **References**

Berg, G., Roskot, N., Steidle, A., Eberl, L., Zock, A., and Smalla, K. (2002) Plant-dependent genotypic and phenotypic diversity of antagonistic rhizobacteria isolated from different *Verticillium* host plants. *Appl Environ Microbiol* **68**: 3328–3338.

Demarre, G., Guerout, A.M., Matsumoto-Mashimo, C., Rowe-Magnus, D.A., Marliere, P., and Mazel, D. (2005) A new family of mobilizable suicide plasmids based on broad host range R388 plasmid (IncW) and RP4 plasmid (IncPalpha) conjugative machineries and their cognate *Escherichia coli* host strains. *Res Microbiol* **156**: 245–255.

Evans, T.J., Crow, M.A., Williamson, N.R., Orme, W., Thomson, N.R., Komitopoulou, E., and Salmond, G.P.C. (2010) Characterization of a broad-host-range flagellum-dependent phage that mediates high-efficiency generalized transduction in, and between, *Serratia* and *Pantoea*. *Microbiology* **156**: 240–247.

Fineran, P.C., Everson, L., Slater, H., and Salmond, G.P. (2005) A GntR family transcriptional regulator (PigT) controls gluconate-mediated repression and defines a new, independent pathway for regulation of the tripyrrole antibiotic, prodigiosin, in *Serratia*. *Microbiology* **151**: 3833–3845.

Matilla, M.A., Espinosa-Urgel, M., Rodriguez-Herva, J.J., Ramos, J.L., and Ramos-Gonzalez, M.I. (2007) Genomic analysis reveals the major driving forces of bacterial life in the rhizosphere. *Genome Biol* **8**: R179.

Matilla, M.A., Fang, X., and Salmond, G.P. (2014) Viunalikeviruses are environmentally common agents of horizontal gene transfer in pathogens and biocontrol bacteria. *ISME J* **8**: 2143–2147.

Matilla, M.A., Stöckmann, H., Leeper, F.J., and Salmond, G.P.C. (2012) Bacterial biosynthetic gene clusters encoding the anti-cancer haterumalide class of molecules: Biogenesis of the broad spectrum antifungal and anti-oomycete compound, oocydin A. *J Biol Chem* **287**: 39125-39138.

McClean, K.H., Winson, M.K., Fish, L., Taylor, A., Chhabra, S.R., Camara, M., *et al*. (1997) Quorum sensing and *Chromobacterium violaceum*: exploitation of violacein production and inhibition for the detection of N-acylhomoserine lactones. *Microbiology* **143**: 3703–3711.

Monson, R., Smith, D.S., Matilla, M.A., Roberts, K., Richardson, E., Drew, A., et al. (2015) A Plasmid-Transposon Hybrid Mutagenesis System Effective in a Broad Range of Enterobacteria. *Front Microbiol* **6**: 1442.

Poulter, S., Carlton, T.M., Su, X., Spring, D.R., and Salmond, G.P.C. (2010) Engineering of new prodigiosin-based biosensors of *Serratia* for facile detection of short-chain N-acyl homoserine lactone quorum-sensing molecules. *Environ Microbiol Rep* **2**: 322–328.

Strobel, G., Li, J.Y., Sugawara, F., Koshino, H., Harper, J., and Hess, W.M. (1999) Oocydin A, a chlorinated macrocyclic lactone with potent anti-oomycete activity from *Serratia marcescens*. *Microbiology* **145**: 3557–3564.
